# Natural Product Synthesis in the 21st Century: Beyond the Mountain Top

**DOI:** 10.1021/acscentsci.3c01518

**Published:** 2024-02-14

**Authors:** Ryan A. Shenvi

**Affiliations:** † Department of Chemistry, Scripps Research, La Jolla, California 92037, United States; ‡ Graduate School of Chemical and Biological Sciences, Scripps Research, La Jolla, California 92037, United States

## Abstract

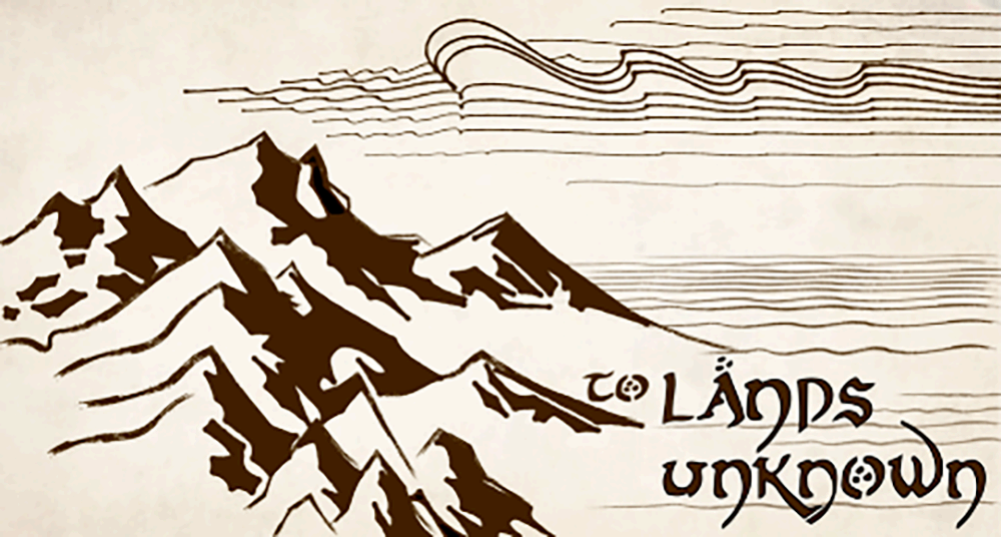

Research into natural products emerged from humanity’s curiosity about the nature of matter and its role in the *materia medica* of diverse civilizations. Plants and fungi, in particular, supplied materials that altered behavior, perception, and well-being profoundly. Many active principles remain well-known today: strychnine, morphine, psilocybin, ephedrine. The potential to circumvent the constraints of natural supply and explore the properties of these materials led to the field of natural product synthesis. This research delivered new molecules with new properties, but also led to fundamental insights into the chemistry of the nonmetal elements H, C, N, O, P, S, Se, and their combinations, i.e., organic chemistry. It also led to a potent culture focused on bigger molecules and races to the finish line, perhaps at the expense of actionable next steps. About 20 years ago, the field began to contract in the United States. Research that focused solely on chemical reaction development, especially catalysis, filled the void. After all, new reactions and mechanistic insight could be immediately implemented by the chemistry community, so it became hard to justify the lengthy procurement of a complex molecule that sat in the freezer unused. This shift coincided with a divestment of natural product portfolios by pharmaceutical companies and an emphasis in academic organic chemistry on applications-driven research, perhaps at the expense of more fundamental science. However, as bioassays and the tools of chemical biology become widespread, synthesis finds a new and powerful ally that allows us to better deliver on the premise of the field. And the hard-won insights of complex synthesis can be better encoded digitally, mined by data science, and applied to new challenges, as chemists perturb and even surpass the properties of complex natural products. The 21st century promises powerful developments, both in fundamental organic chemistry and at the interface of synthesis and biology, if the community of scientists fosters its growth. This essay tries to contextualize natural product synthesis for a broad audience, looks ahead to its transformation in the coming years, and expects the future to be bright.

## Abstract 2

The goal of natural product total synthesis lies beyond target acquisition and in the realm of function. This acknowledgment provides more significance, not less, to the basic science of synthesis.

## Introduction

Mountain climbing as a metaphor for natural product synthesis has been deployed with relish.^1,2,6^ A formidable natural product staggers the mind; its synthesis at once demanding and elegant; a choreography of grips and postures on a cliff face; a struggle to reach the goal no matter the cost. And it is beautiful, a mountain that calls out, “climb me”. Why climb Everest? “Because it is there,” said George Mallory.^3^

But is natural product synthesis this arbitrary? Or inefficient? To journey across mountains, what works better than climbing? Anything. In this spirit, the field of total synthesis has largely moved on from its stereotype, but misunderstanding still dogs the field.

Tracy Kidder’s biography of Paul Farmer, *Mountains Beyond Mountains*,^7^ takes its title from a Haitian proverb that describes how surmounting one problem only leads to the next: *dèyè mòn gen mòn*, “beyond mountains, there are mountains”. Both the terrain of Haiti and its public health problems provide concrete examples. Haiti’s mountains were not items of leisure for Farmer, a physician who founded the charity Partners in Health. Instead, they were barriers that obstructed the delivery of medicine or care to his patients. Haiti exists across the “great epidemiological divide”^7^ that separates rich nations from poor. Its poverty can be traced directly to its history as a former Spanish and French colony composed of lucrative and brutal plantations worked by enslaved peoples of West Africa. A successful slave revolt and defeat of Napoleon’s forces led to the establishment of an independent nation. Yet beyond this mountain, there were more mountains: ostracism of the new Haitian government by Thomas Jefferson’s administration to insulate the United States against similar slave uprisings, and continued threats of reconquest by France, leading to absurd terms of indemnity (including reparations paid to France by former slaves) that left Haiti financially crippled for decades.^8^*Dèyè mòn gen mòn*. Beyond mountains, there are mountains.

It’s fun to climb, and I don’t begrudge the sport. Synthesis is fun too, but it’s not entertainment primarily. Planting one’s flag at the peak does not always signal success, and conquering one mountain after another may not lead anywhere at all. I was asked by the Editorial Board of *ACS Central Science* to describe for a broad audience the future of “target-oriented synthesis”. This is coded language for the total synthesis of natural products, a field spoken of in whispers like a dying relative.^3^ What is it? Why the morbid fascination with its demise? Before we divide the estate, let us consider the promise and trajectory of the field, which can remain strong if problems of real need exist behind the bravura. Here, I will try to describe total synthesis for outsiders. Insiders will notice the absence of case studies or classic syntheses, and I would refer them to outstanding surveys of the field.^5,9,10^This Perspective instead reflects a broad contextualization and an eye on the future. *Dèyè mòn gen mòn*. How can synthesis move beyond proof-of-principle summits up the mountain and into the lands beyond?

It is by no means certain, or even probable, that a compound produced by a microorganism, most likely as a weapon in the struggle for existence, is the very best from the medical point of view. If it is possible to synthesize the compound, it will also be possible to modify the details of the structure and to find the most effective remedies.

Professor A. Fredga, introduction to the 1965 Nobel Prize in Chemistry to R. B. Woodward (ref 11)

## Discussion

Those not involved in chemical synthesis research should consider its widespread and ongoing contributions to society. The field itself resists comprehensive description and consists of the manufacture of polymers, electronics, flavors and fragrances, pigments, and medicine, even if the discussion is restricted to the chemistry of carbon-rich substances (i.e., organic chemistry). Antiquity wielded chemical synthesis without knowing it. For example, indoxyl sulfates secreted by mollusks were aged (dimerized) by heat, light, and oxygen to yield Tyrian purple, a costly fabric dye originating as early as 1200 BC.^12^ Here, we will define **chemical synthesis** (1) as the joining together of molecules^13^ using chemical reactions. This may be contrasted with **degradation** (2) (i.e., the opposite of synthesis,^14,15^) where chemical reactions break down a molecule into smaller parts. Also employed since antiquity, degradation reactions include, for example, the production of soap from animal fats and plant ashes soaked in water (i.e., potash), a description of which can be found on an ancient Sumerian tablet from Lagash dating back to ca. 2500 BC.^16^**Biosynthesis** (3), in contrast to chemical synthesis, occurs within a cell. Biosynthesis benefits from compartmentalization, high local concentrations and pathway redundancy.^17^ It also supplies many building blocks for chemical synthesis. Biosynthesis can be limited, however, by large quantities of extraneous material that must be purified from desired products, and by little control over the diversity of products available. **Self-assembly** (4) can be considered a type of synthesis, but involves the spontaneous aggregation of structures into larger, well-defined products often held together by weak bonds and dictated by equilibrium.^18^ In all cases, the demarcation between terms (1)–(4) is blurry; each feature as aspects of some syntheses.

**Target-oriented synthesis**, as referred to by the Editorial Office, specifies a seemingly obvious feature of synthesis: i.e., you are trying to make something. So, why the special term? *Target*-orientation differentiates itself from other goals of chemical synthesis codified in the literature: **diversity** (diversity-oriented synthesis),^19^**function** (function-oriented synthesis),^20^**biological** relevance,^21^ ease of **purification**,^22^ access to a **general type** of structure (scaffold-oriented).^23^ Overall, I uncovered nine examples of  synthesis, beginning with “biologically oriented” organic sulfur chemistry in 1970.^21^ The frequency of usage increased in the early 2000s^24^ as chemical synthesis adapted to emerging goals in science. These new concepts in synthesis may have been reactions to the emerging predominance of combinatorial chemistry in drug discovery—a randomized mix-and-match approach to synthesis that some have interpreted as writing Shakespeare with a million monkeys on a million typewriters^25^—concomitant with a disinvestment in natural products, which had otherwise served as outstanding sources of new drugs.^26^**Diversity-oriented synthesis** made the case for a compromise of sorts, where the advantageous properties of natural products, especially complexity (see below), might be incorporated into structurally diverse molecular libraries; phenotypic screens might then identify promising leads with new mechanisms of action, analogous to the evolution of function in living systems.^27^ This approach stood in contrast to high-throughput, target-based screens using combinatorial libraries, which tended to be homogeneous, simple, and “flat” (also see below). Diversity-oriented synthesis required a different approach to teaching students: a synthesis designed to target maximally diverse collections would look different than one designed to target a single molecule or its analogues.^27,28^

**Figure 1:**
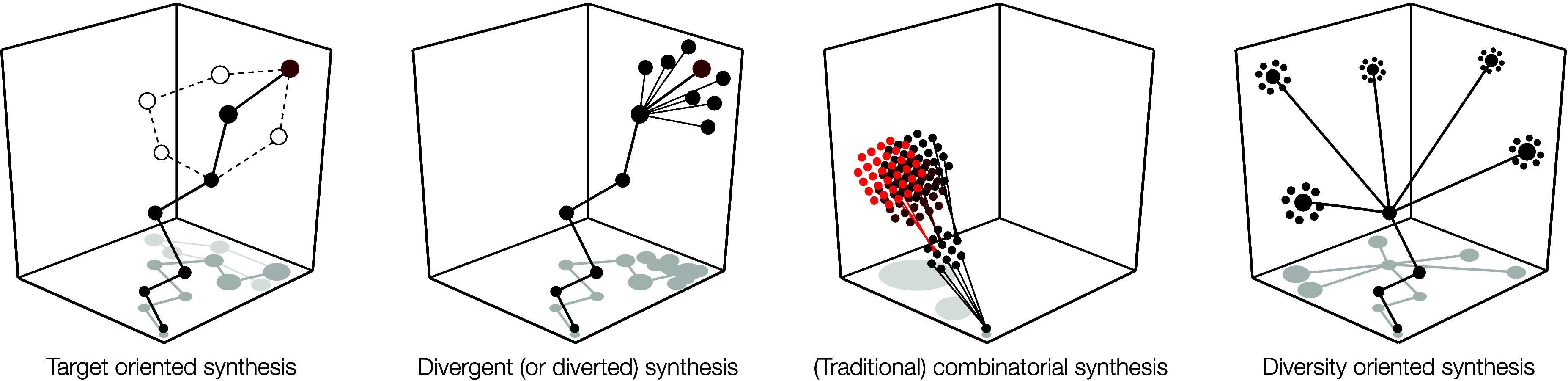
Purpose dictates strategy: types of syntheses. These cartoons depict a chemical space where axes are molecular parameters, points indicate molecules, and lines depict chemical reactions. **Target-oriented** synthesis (or total synthesis) identifies a single molecule for synthesis and navigates challenges in reactivity to reach this goal, much like climbing a mountain. Open circles and dashed lines depict failed routes, which often occur with dense bond networks that complicate synthesis. **Divergent** or **diverted** syntheses will use or design a route to reach analogues of a target; consideration of how and where to diversify a structure will affect route design. **Combinatorial synthesis** seeks to make collections of molecules (molecular libraries) for high-throughput screening. In traditional libraries, these tend to be large numbers of closely related structures, i.e., nondiverse. **Diversity-oriented synthesis** seeks to access broadly distributed collections of small molecules, inspired by the importance of diversity in the evolution of function.^29^

So, although each of these terms sees varying use in the literature, each highlights the idea that purpose dictates strategy (see Figure 1): *why* you approach the mountain affects *how* you approach the mountain.

The foregoing categories of synthesis involve **organic chemistry**, in which **targets** contain only a handful of elements: C, H, O, N, S, P, and the halogens. Modern use of the term “**organic**” may be considered an anachronism, a throwback to vitalism, the idea that living matter consists of a different substance than nonliving matter. Vitalism no longer holds sway, but it had a point. Considering all possible combinations of all atoms, life as we know it consists of, on average and by weight, a narrow range of molecules built from just a few elements.^30^ The choice few atoms of organic molecules, nevertheless, combine in many ways. A well-known estimate from Ciba-Geigy (now Novartis) ballparks the possible combinations of “organic” molecules with ≤30 atoms at 10^60^ members.^31^ Expand these narrow constraints of atom identity or number and you exceed the estimated number of atoms in the universe (ca. 10^76^).^32^

The most fundamental and lasting objective of synthesis is not production of new compounds, but production of new properties.

George S. Hammond, Norris Award Lecture (1968) (ref 33)

**Figure 2:**
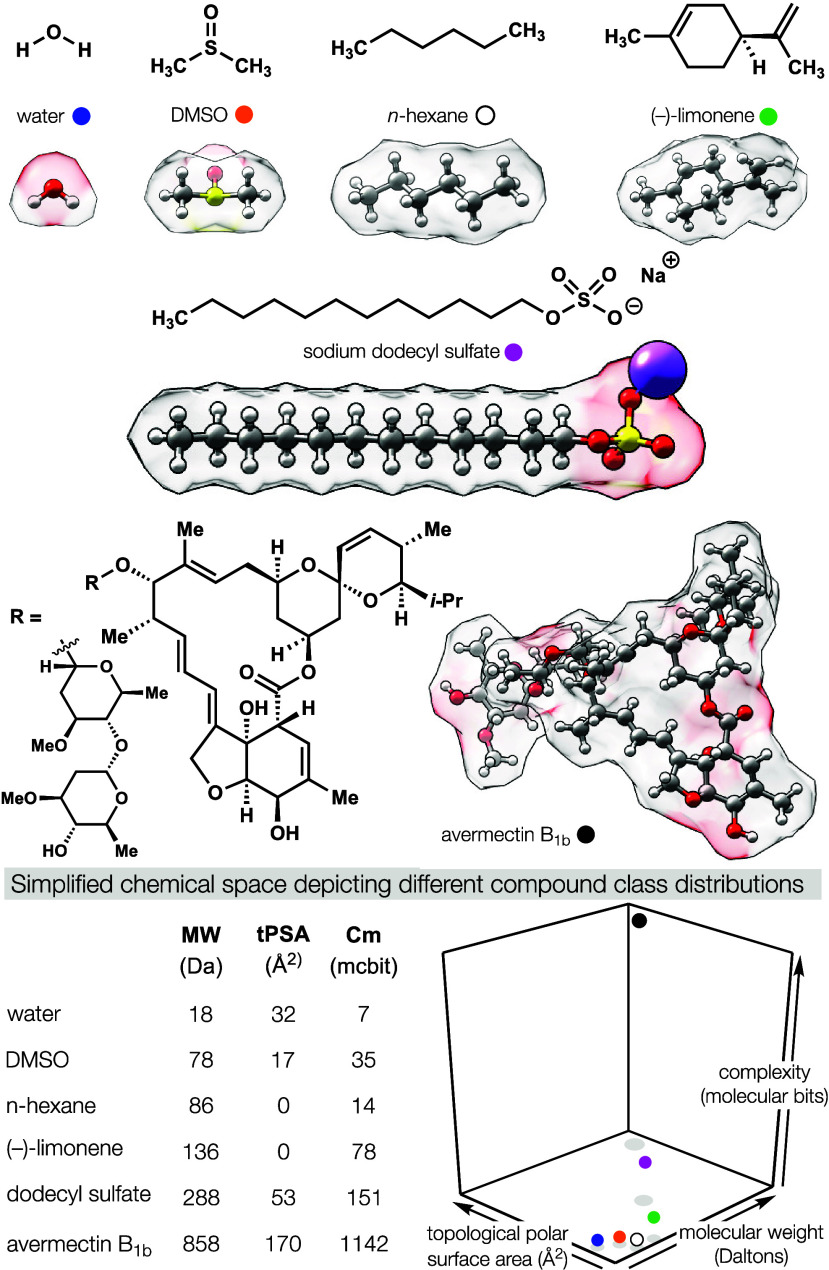
Structure dictates function. Molecules can be parametrized and their properties can be plotted to lie in different regions of a “chemical space”.

This theoretical collection of **organic molecules** contains subdivisions that share similar **molecular properties**: similar atomic content, connectivity, molecular weight, polarity, shape, etc. These properties correlate to function. Detergents tend to be high molecular weight, charged, and nonvolatile; fragrances tend to be lower in weight, uncharged, and volatile. If these molecular properties are used as parameters to construct a coordinate system, you would say that detergents and fragrances reside in different regions of **chemical space** (see Figure 2). The different synthesis strategies shown in Figure 1, for example, are depicted in such a chemical space where axes could correspond to size, weight, shape, surface area, complexity, atom identity, connectivity, and innumerable other parameters. Depending on the type of target molecule, these regions can be far apart or proximal or overlapping. The avermectins are antihelminthics produced by *Streptomyces avermitilis* bacteria and reside in **natural product (NP) space**. They are also FDA-approved drugs and, as polyketides, share structural motifs with many FDA-approved antibiotics. Therefore, if we were to define **drug space** as the molecular properties of all molecules approved for treatment of human disease, some part of this space would reach toward the properties of natural products, as analyses from academia and industry have indeed found.^4^ Independent of natural origin, drugs and natural products share many properties. This is no coincidence: **natural products** control cellular function in the same way that drugs do.

The term “**natural product**” might include all products of metabolism, but commonly refers to a **secondary metabolite**. These are small molecules that lead to increased fitness of closely allied organisms^29^ in an ecological niche. In contrast, a **primary metabolite** is required for normal function among diverse organisms (amino acids, fatty acids, etc.). The category of primary versus secondary metabolite reflects a continuum of structures, but the distinguishing feature of a secondary metabolite is its specialized ecological function.

Secondary metabolites can be considered to be the drugs of nature, often produced by one organism to act on another. As a result, the molecular properties of natural products have been refined by natural selection pressures to yield, on average, aqueous solubility, membrane permeability/transport,^34,35^ biomolecular compatibility (a type of selectivity) and stability. Without these properties, an antibiotic, for example, might not diffuse to a target bacterium and reach its macromolecular target. Or a dart frog toxin might not traverse the gut of a predator and disable its nervous system. Or a mushroom metabolite like psilocybin might not absorb via oral administration penetrate the blood-brain barrier and affect human cognition. Consequently, natural products have been featured as essential medicines in diverse societies throughout history. Their effects on human function can be deliberate or coincidental. That is, a human biomolecule might share close homology and function as the intended target of a plant metabolite or the small molecule might just happen to fit an unrelated binding pocket. Either way, use in historic and current human medicine requires molecular properties that allow access to a cellular target. In other words, many secondary metabolites (natural products) are located in regions of chemical space that tend to allow oral absorption, distribution through the body, and selective biomolecular target binding.

Selection for these same properties occurs in modern drug development. After identification of a small molecule “hit” in a bioassay, the medicinal chemist must design, synthesize, and assay hundreds of small variations of structure that improve on-target function (**pharmacodynamics**)^36^ and other in vivo properties (**pharmacokinetics**)^37^ that separate this hit from a clinical candidate. These properties are hard-won: a game of synthesis whack-a-mole. Improve metabolism and aqueous solubility decreases, improve solubility and binding affinity declines, and improve affinity and exposure plummets. A medicinal chemistry campaign is a creative balancing act, as the team navigates its way through chemical space to find an appropriate candidate. However, given the sheer size of chemical space, even the hundreds of structure modifications represent a narrow “local minimum”. In the grand scheme of things, the drug candidate ends pretty close to where the hit began.^38,39,48^ Therefore, the location of the “hit” in chemical space matters: it will dictate many aspects of a drug’s structure and properties.

So where do **natural products** fit into modern drug discovery? That is a question of ongoing analysis with important consequences for drug design.^40,41^ One significant area identifies NPs as substrates for solute carrier/transporter proteins to explain how NPs reach cellular targets even when their properties lie outside the size/lipophilicity range that describes synthetic drugs.^34,40,40^ The suitability of NPs to interact with biosynthetic proteins, transport proteins, and target proteins suggests that they embody properties that may be superior to strictly synthetic collections—and it recommends the inclusion of these motifs in molecular libraries.^76,40^ There remains, however, the question of procurement and optimization for individual hits. **Phenotypic screens** often identify metabolites with therapeutic properties of promise that lead to new cellular targets, new binding sites, or novel pharmacology.^41^ But after the initial discovery phase, the question becomes, “what next?” The pros and cons of the deployment of natural product leads in medicinal chemistry are dictated by their **physiochemical properties** and **structural complexity**. As mentioned above, the properties of natural products, on average, lend themselves to drug development due to alignment with common medicinal chemistry goals. If, however, a natural product falls outside this average or its pharmacodynamics requires significant change, a cost-benefit analysis must evaluate the feasibility of optimization given the **complexity** of the compound.

**Complexity** can be defined by topology (atom connectivity), stereocenter content, and heteroatom distribution: all of which are inherent features of a molecule with important consequences for selectivity of binding.^42^ This can be confusing to a synthetic chemist (like me) who thinks about how to make a structure, not what information it carries and considers more complex structures harder to make. That is not always true. We see this cognitive dissonance reflected in a fascinating study that correlated knowledge of an existing synthesis to a synthetic chemists’ perception of complexity: how a molecule is made affects how complex we perceive it to be.^43^ Here, however, we will define complexity as **structural complexity**, a measure independent of the synthesis difficulty. There are different ways to define complexity; I favor Böttcher’s additive information content where each atom—its valence, stereochemical content, and connectivity—contributes information, like bits of data.^44^ These sums can be done using a pencil and paper, or via an open-source SMILES string-based calculator.^45,79^ Scores can be averaged across a single molecule (by the number of atoms) or volume of space (expressed in terms of Å^3^) to identify how densely the molecular information is packaged.^79^

**Figure 3:**
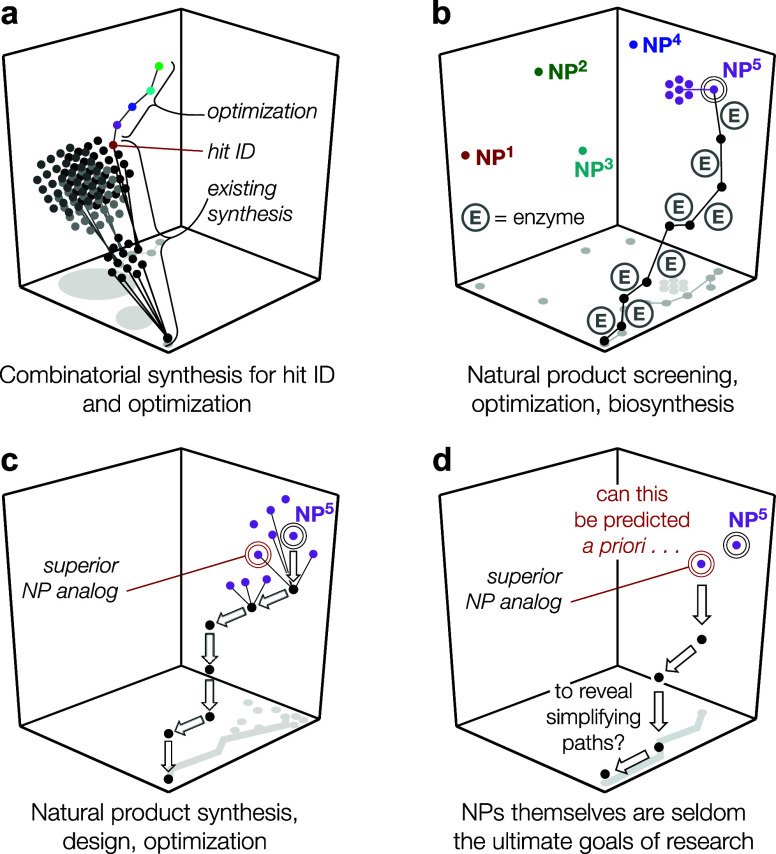
Identification and optimization of hits^50^ from synthetic molecular libraries (a nondiverse case, panel (a)) versus Nature (panel (b)), itself a molecular library. NPs already possess a synthesis (a biosynthesis), but diversification can be constrained by the identification of each enzyme, which must be adapted to suit structural analogs. Chemical synthesis (panel (c)) can identify routes to access and diversify a natural product scaffold to arrive at a superior lead, a mountain beyond the initial mountain of the NP itself. Can these superior analogues (panel (d)) be identified a priori?

Informatics analyses of approved drugs have supported the intuitive view that, on average, drugs tend to be less complex than natural products (this is changing, however; see below). Over the past three decades, drugs have tended to have fewer ring fusions, fewer stereocenters,^46^ fewer H-bond donors/acceptors, and a lower content of sp^3^ atoms than NPs.^46,47^ Independent of structural complexity, drugs appear to be more modular. This also is no coincidence. Because of their origins, drugs represent a compromise between advantageous pharmacokinetic properties, embodied by natural products, and the accessibility of commercial building blocks, which can be sourced inexpensively, exchanged, and modified quickly. Furthermore, many “hits” begin with combinatorial libraries assembled from modular building blocks.^48^ The simplicity and versatility of modular chemistry, combined with the pre-existing synthesis, allows rapid progression to pharmacodynamics and pharmacokinetics goals (see Figure 3a).^49^ Every NP also benefits from an existing synthesis, its biosynthesis, but the enzymes that govern the synthesis are seldom adaptable to divergency (contrast to Figure 3b); i.e., each enzyme must act on a unique substrate. Therefore, the final target can be hard to explore at all its positions, limiting the optimization. If a total synthesis can be designed, then there are more opportunities to optimize and identify a superior compound (Figure 3c). Often this is the main justification for developing a synthesis: control of every position with precision so that a superior compound may be identified.^88^ A question that consumes our laboratory is whether such an analogue may be identified a priori (Figure 3d): why make the natural product if you are planning to not make the natural product? This cynical way of thinking has important consequences to synthetic design because it can alter retrosynthetic pathways.^60^

It is important to note that the complexity-divide between natural product and drug space has begun to change.^51^ Drugs are becoming larger and more complex,^52^ driven by shrinking intellectual property space around common targets,^53,54^ broader/flatter binding sites of interest^55^ and the stringent demands of specificity,^42^ especially for chronic disease. The value of sp^3^-hybridized atoms, stereocenters,^56^ and rigidity^57^—all distinguishing features of natural products—are becoming embraced among some drug hunters to address IP coverage, affinity, and specificity.^52^ These changes cause the gap between druglike and natural product-like space to shrink or blur—good news for academic organic chemistry, as new tools and strategies will be required to solve synthesis problems.

The physicochemical advantage of natural product space^40^ provides an opportunity to regain lost momentum, if the community takes the basic science of synthesis and its translational potential seriously. Can we provide the infrastructure to allow researchers to explore the best ascent up the mountain and not merely plant their flag at the top? Will we also allow researchers to explore the mountains beyond the mountains (function beyond mere synthesis), even if that means skirting the arbitrary point in space that denotes a naturally occurring molecule?

There are implications of this perspective for publication and grant review. I could find only a single paper in *J. Am. Chem. Soc.* in the last 8 years that reports a “studies toward” approach to a natural product, i.e., one in which the natural product is not synthesized, but a general route to access the scaffold is reported.^58^ Historically, this has been mainstream. The absence of these studies in a flagship chemistry journal suggests that the community requires, at least, access to a natural product to establish significance, even if the natural product itself is never again used for research. Why the arbitrary goal line? It also suggests that the introductory paragraph, which usually relies on biomedical relevance of the NP to establish significance, does not consider that Nature’s selection pressure differs from drug optimization.^59^ I do not think this should raise the bar for publication and force synthetic chemists to run bioassays. In fact, the opposite is true: recognition that the local chemical space of a natural product can prove superior to the natural product itself^60b^ should refocus attention on the importance of great approaches to unsolved problems, and re-establish “studies toward”—the basic science of synthesis—as valuable reports in themselves. I suspect that the “death wish” upon the field of total synthesis stems from many exceptional chemists who reflect on their years of graduate school with appreciation for their training but ultimate uncertainty about its value to science, especially given the field’s reputation for extremes. A clear question—of structure, function, or both, associated with target access—might reattract thoughtful students interested in enabling science. These issues, however, are cultural issues. Technology may play a more significant role in the coming years.

### Falling on the Futurism Grenade

Traditional natural product synthesis relies on Nature to identify a target for synthesis. Its selection is justified frequently by its biological function, combined with its structural challenge. Whereas neither are arbitrary per se, the foregoing discussion shows the weakness in these justifications: if function is king, why pursue a structure optimized for survival of the producer organism? If the challenge is structural, why not invent your own challenge? Perhaps we should allow some flexibility to the rigid dogma of the field. If the target is instead viewed as a constellation of structures in the same region of chemical space (see Figure 3), then one can play with structure, function, and synthetic paths according to one’s own goals. Synthetic chemists are not beholden to biosynthesis or evolutionary selection pressures.

As outlined previously,^60^ treatment of a NP target as malleable (or “dynamic”), instead of inalterable (or “static”), allows the chemist to make small changes that maintain the properties of the natural product but alter potential syntheses in enabling ways. The main hurdles that separate this dreamy vision from reality require technological innovations that impact multiple subdisciplines: **predictability, accessibility**, and **automatability**. These are not mature developments, but they are coming.

The counter argument that structure modification results in unpredictable NP function is too narrow of a view. The smallest changes like isotopic substitution (H to D) would be unlikely to change pharmacodynamics.^61^ Modest mutations like heteroatom interchange,^62^ methyl deletion,^60a^ or methyl addition^60d^ can often affect binding, but seldom ablate it completely.^63^ Besides, any affinity loss can be re-established by additional structural changes made possible by synthetic access.^64^ More substantial NP changes like truncation^65,66^ or fragmentation^67^ can be surprising and illuminating; each new piece of data can teach us something. As in silico modeling and docking advance, substantial NP modifications will begin to fall within the realm of prediction.^68^

Of course, this analysis assumes an ideal scenario where a protein target is known, its structure is known, the ligand binding pose is known, and a predictive binding model exists that is consistent with experimental structure–activity relationship (SAR) data. Only a small fraction of NPs will fulfill all of these criteria. But the arrival of AlphaFold^69^ and its ongoing iterations may provide useful protein structures from primary sequences, so that protein selectivity data derived from proteomics experiments may provide some starting point for binding hypotheses. A major question is whether molecular dynamics and in silico docking will improve to the point that actionable confidence can be put into their predictions, especially in the hands of a nonexpert. Few will say that the technology has arrived, but can we claim that it never will? If a predicted protein structure can be used to accurately predict a high affinity binding site and pose of a small molecule, it follows that the relative binding affinity SAR will emerge from iterative predictions. The further layers of functional potencies in the absence of experimental data—i.e., perturbations of protein structure and its effects on complexation/decomplexation, catalysis, and localization, will require decades-more work.

Prediction means nothing in the absence of experimental data. Fortunately, diverse biological assays have become available to researchers outside tightly integrated pharmaceutical teams, so that “real time” analyses of newly synthesized compounds may approach common practice in the coming years.^70^ Cytotoxity assays were not out of place in a *J. Am. Chem. Soc.* total synthesis article (full paper) 20 years ago^71^ and medicinal chemistry programs have existed for decades in academia.^62,67^ Now, however, it is becoming commonplace to find target identification in the same paper as a complex total synthesis campaign^72,73^ or preliminary SAR associated with functional assays like kinase inhibition.^74^ As pointed out by Schreiber,^70^ Anne Carpenter’s “cell painting” method^75^ allows collections to be profiled in a high-throughput, multiplexed way to identify phenotypic changes associated with compounds, independent of any a priori expectation of activity. A study by Waldmann et al. on assembly of a pseudonatural product (PNP) fragment-combination library and screening by cell painting provides a powerful proof-of-principle.^76^ As proteome-wide profiling methods with minimally functionalized or native (nonfunctionalized) compounds advance in practicality and become less expensive,^77,78^ natural product synthetic intermediates and analogues may allow ready identification of highly complex new scaffold classes associated with biological targets of interest. Like advances in analytical techniques can accelerate chemical reaction discovery in the context of total synthesis,^79^ the availability of bioassays may help synthesis campaigns rapidly advance to the mountains beyond. The odds may be increased by deliberately designing the synthesis around a presumed pharmacophore^80,81^ or quickly penetrating predicted bioactive space,^82^ independent of a naturally occurring structure.

**Figure 4:**
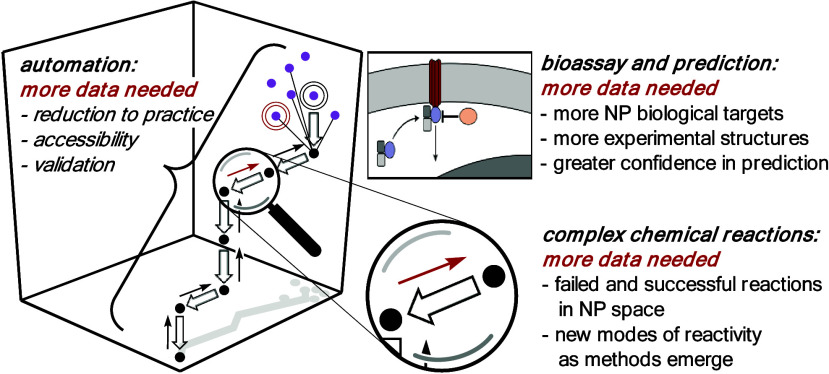
Warning! More data needed at every stage of design: functional models, synthesis routes, and individual reactions!

If prediction of target engagement becomes accurate and if experimental validation becomes widespread (these are big ifs), it follows that automation could accelerate the identification of potent and selective chemical matter, including natural products.^83^ The weak link here is procurement of materials through isolation^41,84^ and synthesis. There is great promise in computational guides to accelerate natural product synthesis: retrosynthetic analysis, transition state analysis, and reaction parametrization. Each of these areas, however, require data^85^ and data require practitioners to explore natural product space (Figure 4). The declining number of groups that pursue fundamental research in multistep synthesis should raise red flags for champions of computer-driven acceleration of synthesis, which lives or dies on the availability of data: reactions that work, reactions that do not work, and a diversity of substrates that show the effect of structure variation on reaction success under different reaction conditions.^86,87^ The same is true of binding models: the community needs more studies of natural product analogs and their SAR to build and validate predictive models of biomolecule liganding. If affinity and synthesis design become aided by prediction, the community can spend less time recapitulating what Nature has already made and more time surpassing it.^88^

## Conclusion

The time is ripe for the new generation of chemists to look at natural products through a different lens, not as mountains to be conquered but as passages to be opened. If the goal of synthesis lies beyond the mountain, then why target a single peak and stop there? Or why not design and target a non-natural analogue superior to the isolated compound?^89,90^ There is so much to discover in natural product chemistry, and at its natural intersection with biology, but both will stagnate without the motive force of the other. The chemistry purists out there who want their natural products independent of biology might consider the contradictory nature of that view. The biological purists who take chemistry for granted might consider the staggering success of chemical biology. Undoubtedly, failure rates will be higher and success slower if access to a target structure signals the beginning, not the end, of a synthesis project. After all, clearing trails receives less acclaim than planting a flag. But, in the long run, the unglamorous work of making a natural product functionally superior, instead of reaching it first, fast, or even best, may provide the field greater significance in the rapidly changing science landscape of the 21st century. The idea of a “supernatural product”^88,91^ has been the basis for the field since its inception.^11^ Now, however, the emerging tools of bioassay and computational prediction, combined with the fundamental science of synthesis, place these once-distant mountains closer than ever before.
